# The Combined Double‐Orifice and Single‐Patch Technique for Partial Atrioventricular Septal Defect in Adults: A Novel Strategy

**DOI:** 10.1155/cdr/8493694

**Published:** 2026-02-24

**Authors:** Hua-Jie Zheng, Rui-Han Xiao, Mao-Ting Ye, Jun Li, Mei Guo, San-Jiu Yu, Yong-Bo Cheng, Wei Cheng

**Affiliations:** ^1^ Department of Cardiac Surgery, Southwest Hospital, Third Military Medical University (Army Medical University), Chongqing, China, tmmu.edu.cn

**Keywords:** double-orifice repair, partial atrioventricular septal defect, single-patch technique, tricuspid valve dysplasia

## Abstract

**Aim:**

The aim of this study is to share our experience of the combined double‐orifice and single‐patch technique for the correction of partial atrioventricular septal defect (pAVSD) and its associated tricuspid valve dysplasia (TVD).

**Methods:**

Between January 2014 and May 2024, 99 patients (age ≥ 18 years) who underwent repair of pAVSD and its associated TVD using the aforementioned strategy were retrospectively analyzed.

**Results:**

The mean aortic cross‐clamp time was 48.5 ± 12.6 min, and the mean cardiopulmonary bypass time was 75.8 ± 13.0 min. Follow‐up was 100% at a mean of 5.2 years (3–8 years). No operative or late deaths occurred. No evidence of mitral stenosis was detected, and only one patient developed a severe MR 2 years after the surgery and underwent reoperation. There was no moderate or severe tricuspid regurgitation during follow‐up. There was neither secondary tricuspid valve repair nor permanent pacemaker implantation among all patients.

**Conclusions:**

The combined double‐orifice and single‐patch technique is a safe, effective, and durable strategy for repair of pAVSD and its associated TVD, demonstrating an excellent technical reproducibility.

## 1. Introduction

Partial atrioventricular septal defect (pAVSD), classically described as an ostium primum atrial septal defect (ASD), is part of the spectrum of endocardial cushion defects [1]. pAVSD constitutes merely 1%–2% of all congenital heart malformations and demonstrates particular rarity in adults [2]. pAVSD is characterized by an ostium primum ASD and a cleft dividing the anterior mitral valve leaflet. The precise incidence of tricuspid valve dysplasia (TVD) in pAVSD is indeed sparsely documented. A study has reported that approximately 80% of the patients have TVD, mostly involving a small septal leaflet [3]. In our clinical experience, echocardiographic and intraoperative findings consistently demonstrate a high prevalence of TVD in adult patients with pAVSD. However, standard treatment techniques for children often fail to provide a lasting solution to these problems.

Inspired by Alfieri′s seminal double‐orifice technique for mitral valve repair in anterior leaflet prolapse [4], we merely adapted this approach to address residual moderate or higher MR following mitral cleft closure in pAVSD. Furthermore, we further developed a simplified single‐patch technique utilizing a single autologous pericardial patch to simultaneously close the ASD and reconstruct the dysplastic tricuspid septal leaflet. This study is aimed at presenting our experience with this combined double‐orifice and single‐patch technique for the correction of pAVSD and its associated TVD in adults.

## 2. Methods

### 2.1. Patients

Between January 2014 and May 2024, 128 consecutive patients (age ≥ 18 years) who had pAVSD and its associated TVD were admitted to our institution for surgical treatment. It is important to realize that chronic mitral regurgitation not only results in degenerative changes in the leaflets and supporting chordae but also leads to annular dilation. Therefore, in some cases, in addition to closing the cleft, an annuloplasty technique is also necessary to stabilize the annulus. However, in our center, only a very small number of patients have received annuloplasty. For those patients who have had the repair of the anterior leaflet of the mitral valve but still have moderate or higher regurgitation, we prefer to adopt the double‐orifice technique. After screening, a total of 99 patients received the double‐orifice repair combined with our single‐patch technique and were retrospectively analyzed.

The study was approved by our institutional ethics committee (Institutional Review Board Approval Number: KA20180106DR1, January 1, 2018) and conducted in accordance with the Declaration of Helsinki (as revised in 2013). Written informed consent was obtained from all study participants.

### 2.2. Echocardiographic Assessment

Echocardiography checkups were performed according to the American Society of Echocardiography guidelines [5]. All examinations were performed by two expert echocardiographers (R‐H.X. and S‐J.Y.) using a commercially available ultrasound equipment (Philips EPIQ CVx, Philips Medical Systems). Valve regurgitation severity was graded by color Doppler echocardiography as 0 (none), 1 (trace), 2 (mild), 3 (moderate), or 4 (severe).

All patients underwent comprehensive transesophageal echocardiography (TEE) both preoperatively for accurate diagnosis of pAVSD and associated TVD, and intraoperatively to assess surgical outcomes. Transthoracic echocardiography (TTE) was routinely performed before discharge to verify treatment efficacy.

Typically, the preoperative diagnosis was as follows: (1) Dysplastic tricuspid valve with hypoplastic/absent septal leaflet and restricted mobility (Abbreviations: MR, mitral regurgitation; NYHA, New York Heart Association; TR, tricuspid regurgitation. S1a),accompanied by tricuspid regurgitation (TR) (Figure S1b); (2) primum ASD with bidirectional shunting (Figure S1c); and (3) anterior mitral leaflet cleft (Figure S1d) with MR (Figure S1e).

### 2.3. Surgical Techniques

We selectively employ the combined double‐orifice and single‐patch technique for patients with pAVSD and significant TVD. The indications for this approach include (1) persistence of moderate or greater MR following initial repair of the anterior leaflet; (2) the presence of a sizable ASD; and (3) moderate or severe TR secondary to maldevelopment of the tricuspid valve′s septal leaflet, which is typically short or absent.

The operation was conducted through a median sternotomy utilizing cardiopulmonary bypass with moderate hypothermia (28°C–32°C), aortic cross‐clamping, and cold blood cardioplegia. Intraoperative examination revealed the following characteristic morphology: severely dysplastic tricuspid septal leaflet with short aberrant chordae inserting directly into the ventricular wall (Figure S2a); a primum‐type ASD; mitral and tricuspid valves sharing a common annular plane; and thickened, curled edges of the mitral valve cleft (Figure S2b). Figure S2c schematically illustrates this anatomical configuration.

#### 2.3.1. The Double‐Orifice Repair to Correct Mitral Regurgitation

Through the defect of the atrial septum, the zone of apposition (ZOA) of the anterior leaflet of the mitral valve was completely closed primarily with interrupted 6‐0 polypropylene sutures (Figure 1a).

Figure 1Left atrioventricular valve repair. The patient′s head is oriented toward the inferior image border. (a) Primary complete closure of the anterior mitral leaflet zone of apposition (white dashed outline). (b) Double‐orifice reconstruction demonstrating edge‐to‐edge approximation of anterior and posterior leaflets (white dashed outline).

**Figure:**
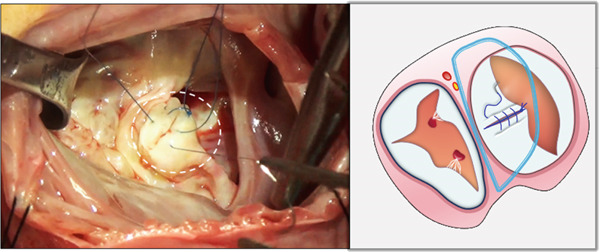
(a)

**Figure:**
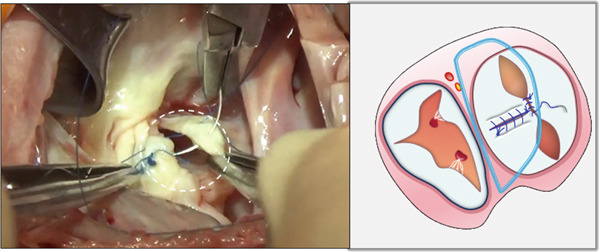
(b)

Based on annular size and saline testing results, we may employ the previously described double‐orifice technique [6] (Figure 1b), which typically includes the entire P2 scallop of the posterior leaflet.

#### 2.3.2. The Single‐Patch Technique to Close ASD and Reconstruct the Septal Leaflet

i. A trapezoidal autologous pericardial patch was carefully harvested (Figure 2a), with attention to maintaining adequate size to allow for final trimming during implantation. The patch was then folded at the one‐third point to create two functionally distinct portions: (1) a smaller segment for tricuspid leaflet augmentation and (2) a larger segment for ASD closure (Figure 2b).
ii. The pericardial patch was secured to the basal portion of the septal leaflet (rather than the annulus) using pledgeted 4‐0 polypropylene mattress sutures placed from the ventricular to atrial aspect. The initial suture was positioned at the septal edge of the anterior leaflet base (Figure 3a), with the final suture placed at the septal‐posterior commissure (Figure 3b). A continuous 5‐0 polypropylene suture line was then created along the coronary sinus orifice margin, maintaining its drainage into the left atrium (Figure 3c).
iii. To optimize leaflet function, two edge‐to‐edge sutures were added between the posterior edge of the residual septal leaflet and the patch (Figure 3d), allowing the patch to integrate with the existing chordal support. In addition, two edge‐to‐edge sutures were also added between the septal edge of the anterior leaflet and the anterior‐side patch (Figure 3e).
iv. The patch apex was securely anchored to a discrete muscular ridge on the interventricular septum using pledget‐reinforced horizontal mattress sutures (Figure 3f). Precise adjustment of the base‐to‐apex length ensured an adequate patch curvature, facilitating optimal coaptation with both anterior and posterior leaflets (Figure 3g).

Figure 2Pericardial patch preparation. The patient′s head is oriented toward the inferior aspect. (a) A trapezoidal autologous pericardial patch was sized to 1.5 times the combined area of the ASD and deficient tricuspid leaflet. (b) The patch was folded at the one‐third position (white dotted line), creating separate portions for septal leaflet augmentation (smaller segment) and ASD closure (larger segment).

**Figure:**
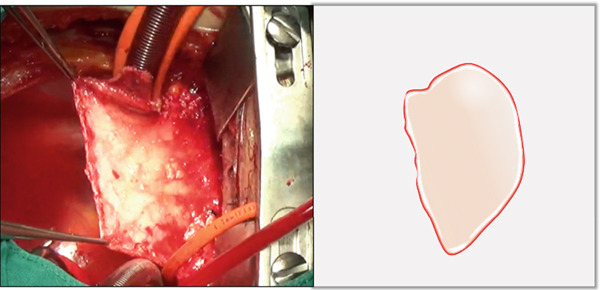
(a)

**Figure:**
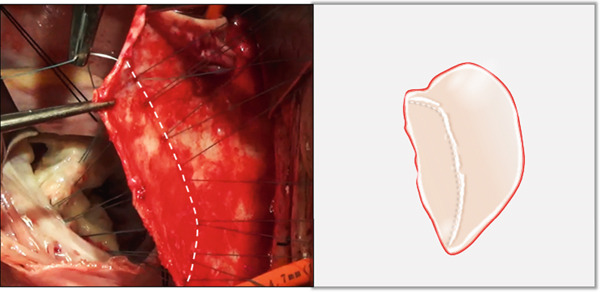
(b)

Figure 3Single‐patch technique surgical steps. The patient′s head is oriented toward the inferior image border. The pericardial patch was secured to the septal leaflet base using the following sequence: (a) Initial suture placement at the anterior leaflet′s septal border (white dashed outline); and (b) Final suture placement at the septal‐posterior commissure (white dashed outline). (c) Continuous 5‐0 polypropylene suture along the coronary sinus margin (white dashed outline), followed by patch trimming and ASD closure. (d) Posterior leaflet‐to‐patch edge approximation (white dashed outline). (e) Anterior leaflet‐to‐patch edge approximation (white dashed outline). (f) Patch apex fixation to the interventricular septum using reinforced horizontal mattresses (white dashed outline). (g) Final patch dimensions ensuring optimal leaflet coaptation.

**Figure:**
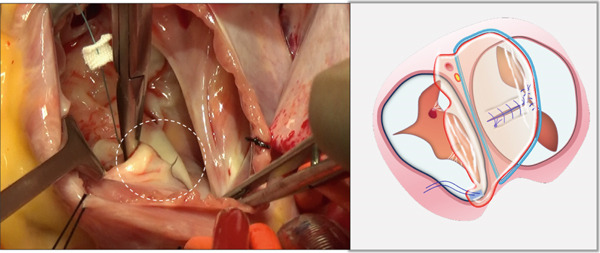
(a)

**Figure:**
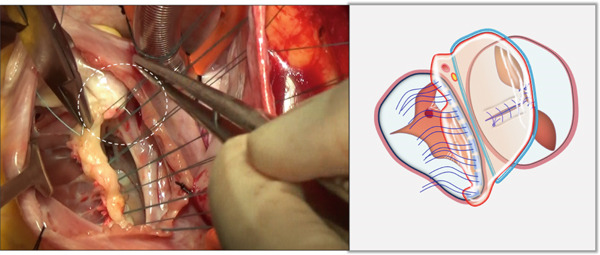
(b)

**Figure:**
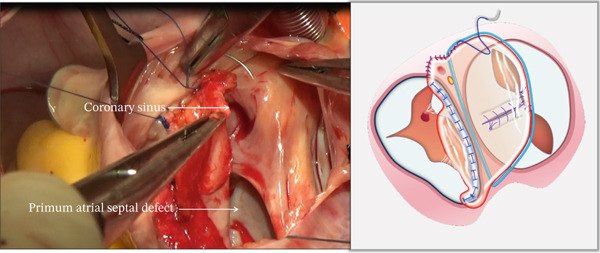
(c)

**Figure:**
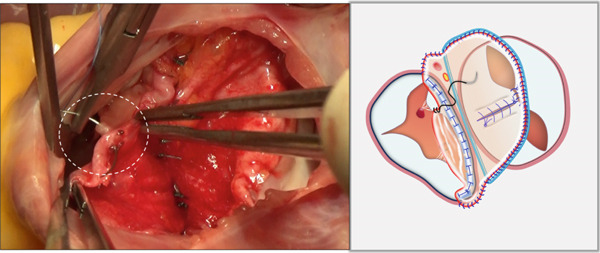
(d)

**Figure:**
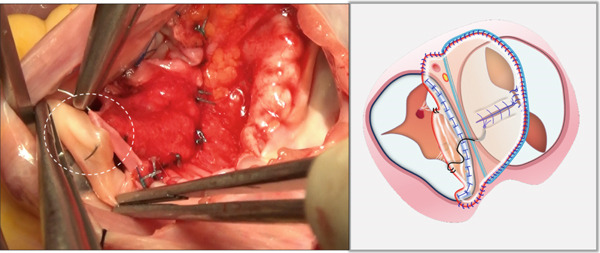
(e)

**Figure:**
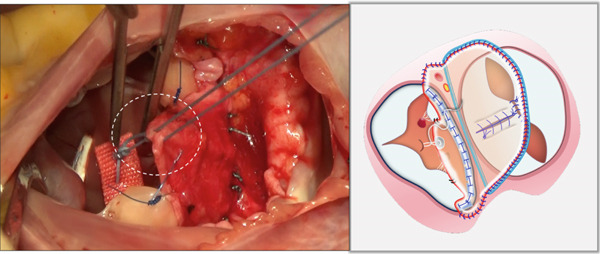
(f)

**Figure:**
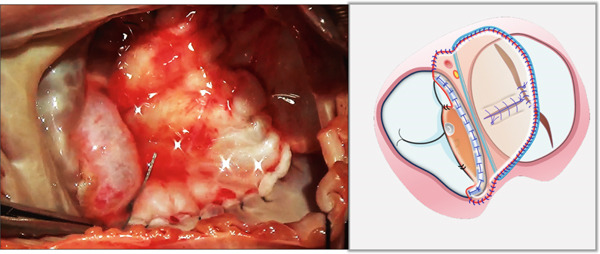
(g)

Repair success was defined by the following hemodynamic criteria: ≤ Grade 2 residual regurgitation in both AV valves, and mean transvalvular gradients < 7–8 mmHg without evidence of functional stenosis. TEE demonstrated: (i) successful ASD closure (Figure S3a); (ii) augmented tricuspid septal leaflet dimensions with absent regurgitation and normal transvalvular gradients (Figure S3b); (iii) edge‐to‐edge mitral valve repair configuration (Figure S3c); (iv) three‐dimensional confirmation of dual‐orifice mitral valve anatomy (Figure S3d); and (v) trace mitral regurgitation (Figure S3e) with a mean transvalvular gradient of < 7–8 mmHg without stenosis (Figure S3f).

### 2.4. Follow‐Up

Postoperative TTE was performed at regular intervals (every 6 months for the first year, then annually). Patients were followed up until the date of the last follow‐up or death recorded (last queried June 5, 2025). Data were collected from patient records or from telephone calls with the patient or the patient′s family. All baseline clinical, procedural, and follow‐up data were entered into a dedicated web‐based database, held and maintained in our institution.

### 2.5. Statistical Analysis

The categorical variables were presented as numbers (percentages) and were compared using a *χ* ^2^ test or Fisher′s exact test. The continuous variables are presented as mean and SD or median and interquartile range (IQR). Based on their distributions, the continuous variables were compared using the student′s *t*‐test or the Wilcoxon rank sum test between two groups. Statistical analysis was performed using SPSS Version 25 (SPSS Inc., Chicago, Illinois). Two‐tailed *p* value < 0.05 was considered statistically significant.

## 3. Results

### 3.1. Patient Demographics and Preoperative Characteristics

The median age at the time of operation was 31.2 ± 10.6 years with 60.6% (*n* = 60) females. Other demographic and preoperative characteristics are shown in Table 1.

**Table 1:** Baseline characteristics.

Variables	All (*n* = 99)
Age at operation (years)	31.2 ± 10.6
Weight at operation (Kg)	53.8 ± 11.5
Female	60 (60.6%)
Preoperative MR degree	
Trace/none	0 (0%)
Mild	0 (0%)
Moderate	30 (30.3%)
Severe	69 (69.7%)
Preoperative TR degree	
Trace/none	0 (0%)
Mild	0 (0%)
Moderate	42 (42.4%)
Severe	57 (57.6%)
Preoperative arrhythmia	
Atrial fibrillation	18 (18.2%)
Atrioventricular Block III	0 (0%)
Bundle branch block	57 (57.6%)
Preoperative NYHA class	
I	12 (12.1%)
II	51 (51.5%)
III	33 (33.3%)
IV	3 (3.0%)

*Note:* Values are *n* (%) or mean ± SD.

Abbreviations: MR, mitral regurgitation; NYHA, New York Heart Association; TR, tricuspid regurgitation.

### 3.2. Operative Details

Operative details are shown in Table 2. The mean aortic cross‐clamp time was 48.5 ± 12.6 min, and the mean cardiopulmonary bypass time was 75.8 ± 13.0 min. Sixty (60.6%) patients underwent De Vega valvuloplasty for residual TR, whereas nine (9.1%) patients received ring annuloplasty. All patients were in normal sinus rhythm at the end of the operation.

**Table 2:** Operative details.

Variables	All (*n* = 99)
Aortic cross‐clamp time (min)	48.5 ± 12.6
CPB time (min)	75.8 ± 13.0
Closure of ostium primum by single‐patch technique	99 (100%)
Secundum atrial septal defect closure	24 (24.2%)
Left atrioventricular valve repair	
ZOA suture closure	99 (100%)
Double‐orifice technique	99 (100%)
Right atrioventricular valve repair	
Septal leaflet reconstruction by single‐patch technique	99 (100%)
De Vega annuloplasty	60 (60.6%)
Ring annuloplasty	9 (9.1%)
Arrhythmia surgery	6 (6.1%)

*Note:* Values are *n* (%) or mean ± SD.

Abbreviations: CPB, cardiopulmonary bypass; ZOA, zone of apposition.

### 3.3. Postoperative Details

Postoperative details are shown in Table 3. No patient required a permanent pacemaker prior to discharge. All patients have been asymptomatic postoperatively, without requiring any medication, including anticoagulants. The mean hospital stay after surgery was 10.0 ± 2.5 days.

**Table 3:** Postoperative and follow‐up details.

Variables	All (*n* = 99)
Length of hospital stay (day)	10.0 ± 2.5
Residual complications	
Atrial fibrillation	0 (0%)
Atrioventricular Block III	0 (0%)
Bundle branch block	33 (33.3%)
Early mortality (within 30 days)	0 (0%)
Late mortality	0 (0%)
Reoperation	
Permanent pacemaker implantation	0 (0%)
Mitral valve repair	3 (3.0%)
Mitral valve replacement	0 (0%)
Tricuspid valve replacement	0 (0%)
Most recent follow‐up heart function	
NYHA Class I	72 (72.7%)
NYHA Class II	27 (27.3%)
NYHA Class III or IV	0 (0%)

*Note:* Values are *n* (%) or mean ± SD.

Abbreviation: NYHA, New York Heart Association.

### 3.4. Survival

There were no intraoperative or late deaths in this series.

### 3.5. Follow‐Up

Complete follow‐up was achieved (100%) at a mean duration of 5.2 years (range: 3–8 years). Postoperative echocardiography was performed in all cases. No mitral stenosis was observed. Specifically, the mean mitral valve area, measured by planimetric 2D echocardiography, was 2.6 ± 0.55 cm^2^ and remained stable over time. No patient developed significant transvalvular gradients (> 8 mmHg) during follow‐up. Preoperative versus postoperative MR grades demonstrated significant improvement (*p* < 0.001) (Figure 4a). Only one patient with an initially uncomplicated postoperative course (discharged postoperative Day 9) developed a late valve prolapse with severe MR due to chordal elongation at 2 years. Following reoperation, she maintains excellent clinical status with less than moderate MR and no stenosis. There was no moderate TR after surgery (*p* < 0.001), which was sustained across follow‐up with no detectable evidence of change (*p* = 0.628) (Figure 4b). There was neither secondary tricuspid valve repair nor permanent pacemaker implantation among all patients (Table 3).

Figure 4Longitudinal valve function assessment. (a) MR severity progression during follow‐up. (b) TR severity progression during follow‐up. MR, mitral regurgitation; TR, tricuspid regurgitation.

**Figure:**
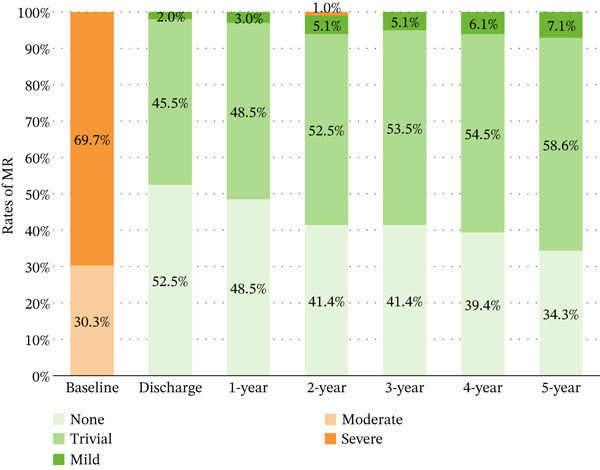
(a)

**Figure:**
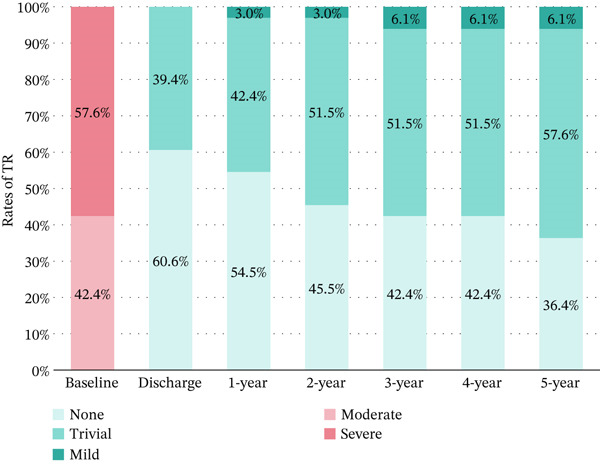
(b)

## 4. Discussion

To simplify the surgical procedure and achieve excellent outcomes, we implemented a combined double‐orifice and single‐patch technique. From January 2014, this strategy was routinely applied in 99 adult patients with these defects. Our results demonstrated favorable short‐term outcomes with minimal reoperation requirements.

In adult pAVSD, the mitral valve demonstrates both leaflet abnormalities (including characteristic clefts along the free edge, particularly in the anterior leaflet) and annular dilation/distortion [7, 8]. Consequently, annular stabilization via annuloplasty is typically required in addition to cleft closure. However, we routinely employed the central double‐orifice repair to treat residual MR after closing the cleft due to its technical simplicity and demonstrated efficacy [9]. Of note, a meticulous mitral valve assessment remains essential for optimal leaflet approximation and suture placement. Although achieving complete MR elimination, maintaining adequate valve area is equally crucial. Improper technique application may lead to either residual regurgitation or iatrogenic stenosis. Although all cases demonstrated a reduced mitral orifice area, no hemodynamically significant stenosis occurred in our series.

After the repair of pAVSD, AV block is not uncommon postoperatively, primarily due to the inferior displacement of the AV node near the coronary sinus ostium [10, 11]. The region extending from the coronary sinus ostium to the midportion of the interatrial septum constitutes a high‐risk zone for conduction tissue injury. Consequently, sutures must be placed distant from this area and secured superficially to preserve conduction integrity. There are usually two types of suturing methods [12]: (i) the Kirklin approach, utilizing an oversized pericardial patch to exclude the Koch triangle′s right border while diverting coronary sinus drainage to the left atrium; and (ii) the McGoon technique, involving annular suturing along the left AV valve and left atrial wall, maintaining coronary sinus drainage into the right atrium. Our single‐patch technique anchored the pericardial patch to the base of the septal leaflet rather than the annulus adjacent to the conduction system, and the coronary sinus was also directed into the left atrium. This strategy successfully prevented both AV nodal injury and interatrial conduction disturbances, evidenced by consistent sinus rhythm maintenance and absence of permanent pacing requirements in all cases.

Although conventional annuloplasty techniques, including ring annuloplasty, De Vega′s annuloplasty, and Kay′s annuloplasty, are beneficial for addressing the annular dilatation associated with functional TR, their efficacy may be limited with markedly hypoplastic leaflets [13]. Leaflet augmentation, although effective for functional TR with a small leaflet, is relatively complex and time‐consuming [14]. Here, we describe a simplified technique that simultaneously closes the ASD and reconstructs the septal leaflet using a single autologous pericardial patch. Specifically speaking, we reserve a portion of the pericardium on the right side of the ventricular septal crest and interruptedly sutured it to the basal part of the septal leaflet to enlarge the septal leaflet. Compared with traditional leaflet augmentation, our technique allows patch augmentation of the septal leaflet in cases with small septal leaflets and also offers simplicity due to fewer sutures. Long‐term follow‐up is needed to evaluate the durability of the reconstructed septal leaflet.

Overall, this technique was developed to address the adult pAVSD subgroup with a concomitant primum ASD and a short, dysplastic tricuspid septal leaflet—a scenario where standard multipatch repairs can be technically complex. The proposed mechanism utilizes a single pericardial patch to achieve an integrated repair: it closes the ASD while simultaneously, via a tailored extension, lengthening the deficient septal leaflet to restore valve competence. This approach embodies the principle of “anatomic simplification through functional integration.”

### 4.1. Study Limitations

Given the limited durability associated with our historical results using traditional multipatch or ring annuloplasty‐based strategies, our practice has evolved away from these techniques, and a comparative analysis was not feasible due to the small cohort size. This was a retrospective study with a relatively small sample size and the data obtained from a single‐center clinical practice. The follow‐up period was relatively short. Larger multicenter studies with extended surveillance are warranted to validate the advantages of the combined double‐orifice and single‐patch technique.

## 5. Conclusions

The combined double‐orifice and single‐patch technique is a safe, effective, and durable strategy for repair of pAVSD and its associated TVD, demonstrating an excellent technical reproducibility. The outcomes were satisfactory in the short term, with minimal reoperations. It could be used as an alternative technique for adult patients with pAVSD and its associated TVD.

**Nomenclature**
ASD: atrial septal defect
MR: mitral regurgitation
NYHA: New York Heart Association
PAVSD: partial atrioventricular septal defect
TR: tricuspid regurgitation
TVD: tricuspid valve dysplasia

## Author Contributions

Y‐B.C. and W.C. were responsible for the study concept and design. M.G. and S‐J.Y. were responsible for the acquisition and analysis of data. All authors contributed to the interpretation of the data. J.L. developed the figures. H‐J.Z., R‐H.X., and M‐T.Y. drafted the manuscript. The corresponding authors attest that all listed authors meet authorship criteria. H‐J.Z., R‐H.X., and M‐T.Y. contributed equally to this work.

## Funding

This study was supported by Chongqing Natural Science Foundation (CSTB2023NSCQ‐ZDX0014 and CSTB2023NSCQ‐MSX0672).

## Disclosure

All authors approved the final version and agreed to be accountable for all aspects of the work.

## Ethics Statement

The study was approved by our institutional ethics committee (Institutional Review Board Approval Number: KA20180106DR1, January 1, 2018) and conducted in accordance with the Declaration of Helsinki (as revised in 2013). Written informed consent was obtained from all study participants.

## Consent

The authors have nothing to report.

## Conflicts of Interest

The authors declare no conflicts of interest.

## Supporting Information

Additional supporting information can be found online in the Supporting Information section.

## Supporting information


**Supporting Information 1** Figure S1: Preoperative transesophageal echocardiographic findings. (a) Two‐dimensional imaging demonstrated complete absence or severe hypoplasia of the tricuspid septal leaflet. (b) Color Doppler revealed mild tricuspid regurgitation. (c) Bidirectional interatrial shunting was evident. (d) Three‐dimensional reconstruction clearly delineated the anterior mitral leaflet cleft. (e) Severe mitral regurgitation was documented. LA, left atrium; LV, left ventricle; RA, right atrium; RV, right ventricle.

**Supporting Information 2** Figure S2: Intraoperative anatomical findings. The patient′s head is oriented toward the image bottom. (a) A hypoplastic but anatomically positioned septal leaflet (white dashed outline). (b) The mitral valve cleft (white dashed outline) retracted through the ostium primum atrial septal defect into the right atrial cavity. (c) Schematic representation of pAVSD morphology featuring a hypoplastic yet normally positioned septal leaflet (SL). pAVSD, partial atrioventricular septal defect.

**Supporting Information 3** Figure S3: Postoperative echocardiographic assessment. (a) Confirmed ASD closure. (b) Augmented tricuspid septal leaflet without regurgitation or stenosis. (c) Edge‐to‐edge mitral valve repair. (d) Three‐dimensional demonstration of double‐orifice mitral valve configuration. (e) Trace mitral regurgitation. (f) Mean transvalvular gradient of 2.8 mmHg. LA, left atrium; LV, left ventricle; MR, mitral regurgitation; RA, right atrium; RV, right ventricle; TR, tricuspid regurgitation.

## Data Availability

The datasets generated during and/or analyzed during the current study are available from the corresponding author upon reasonable request.
