# Supplementary material for: The Combined Double‐Orifice and Single‐Patch Technique for Partial Atrioventricular Septal Defect in Adults: A Novel Strategy
Source: Cardiovasc Ther. 2026 Feb 24;2026:8493694. doi: 10.1155/cdr/8493694 (PMC12932322; doi:10.1155/cdr/8493694)
Supplement: Supplementary file 1 — Supporting Information 1 Figure S1: Preoperative transesophageal echocardiographic findings. (a) Two‐dimensional imaging demonstrated complete absence or severe hypoplasia of the tricuspid septal leaflet. (b) Color Doppler revealed mild tricuspid regurgitation. (c) Bidirectional interatrial shunting was evident. (d) Three‐dimensional reconstruction clearly delineated the anterior mitral leaflet cleft. (e) Severe mitral regurgitation was documented. LA, left atrium; LV, left ventricle; RA, right atrium; RV, right ventricle. [file CDR-2026-8493694-s001.pptx]

## Slide 1
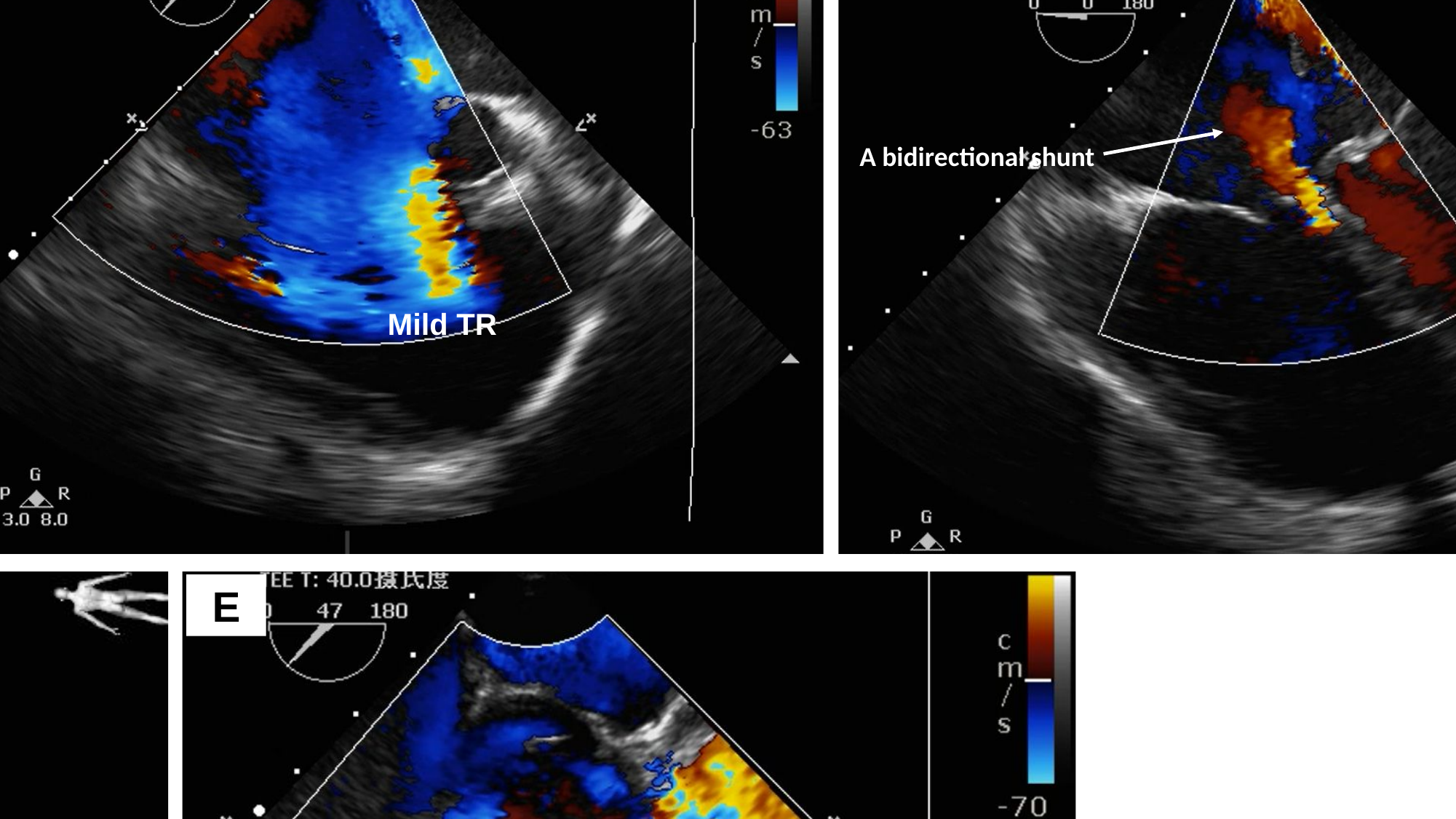

LA
RA
LV
RV
The septal leaflet was severely dysplastic.
A
ASD
Mild TR
B
A bidirectional shunt
C
Mitral cleft
D
Severe MR
E
