# Supplementary material for: The Combined Double‐Orifice and Single‐Patch Technique for Partial Atrioventricular Septal Defect in Adults: A Novel Strategy
Source: Cardiovasc Ther. 2026 Feb 24;2026:8493694. doi: 10.1155/cdr/8493694 (PMC12932322; doi:10.1155/cdr/8493694)
Supplement: Supplementary file 2 — Supporting Information 2 Figure S2: Intraoperative anatomical findings. The patient′s head is oriented toward the image bottom. (a) A hypoplastic but anatomically positioned septal leaflet (white dashed outline). (b) The mitral valve cleft (white dashed outline) retracted through the ostium primum atrial septal defect into the right atrial cavity. (c) Schematic representation of pAVSD morphology featuring a hypoplastic yet normally positioned septal leaflet (SL). pAVSD, partial atrioventricular septal defect. [file CDR-2026-8493694-s002.pptx]

## Slide 1
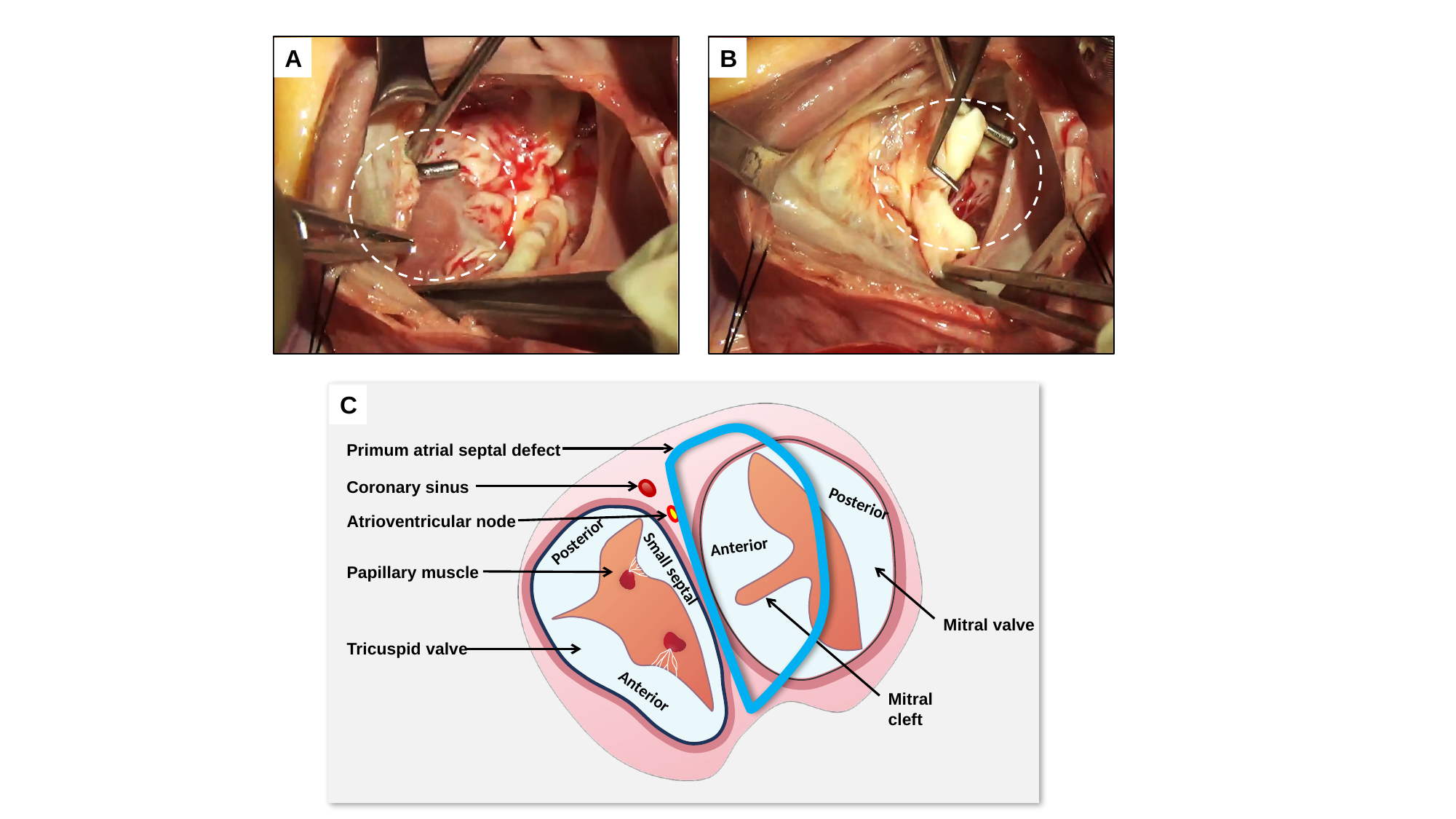

A
B
C
Primum atrial septal defect
Coronary sinus
Posterior
Atrioventricular node
Posterior
Anterior
Papillary muscle
Small septal
Mitral valve
Tricuspid valve
Mitral cleft
Anterior
