# Supplementary material for: The Combined Double‐Orifice and Single‐Patch Technique for Partial Atrioventricular Septal Defect in Adults: A Novel Strategy
Source: Cardiovasc Ther. 2026 Feb 24;2026:8493694. doi: 10.1155/cdr/8493694 (PMC12932322; doi:10.1155/cdr/8493694)
Supplement: Supplementary file 3 — Supporting Information 3 Figure S3: Postoperative echocardiographic assessment. (a) Confirmed ASD closure. (b) Augmented tricuspid septal leaflet without regurgitation or stenosis. (c) Edge‐to‐edge mitral valve repair. (d) Three‐dimensional demonstration of double‐orifice mitral valve configuration. (e) Trace mitral regurgitation. (f) Mean transvalvular gradient of 2.8 mmHg. LA, left atrium; LV, left ventricle; MR, mitral regurgitation; RA, right atrium; RV, right ventricle; TR, tricuspid regurgitation. [file CDR-2026-8493694-s003.pptx]

## Slide 1
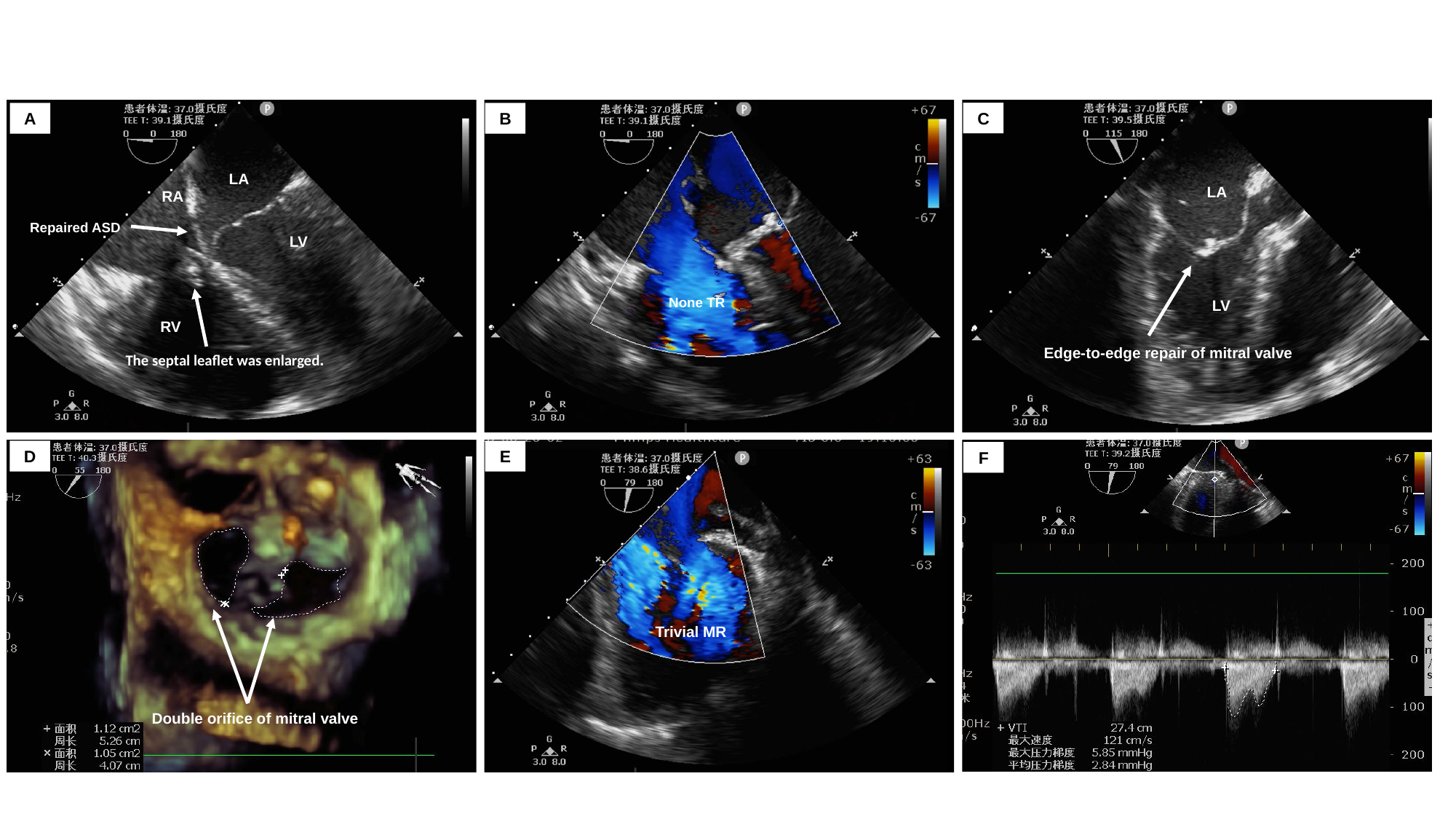

A
LA
RA
Repaired ASD
LV
The septal leaflet was enlarged.
RV
B
None TR
C
LA
LV
Edge-to-edge repair of mitral valve
F
D
E
Trivial MR
Double orifice of mitral valve
